# Lower Extremity Joint Angle Tracking with Wireless Ultrasonic Sensors during a Squat Exercise

**DOI:** 10.3390/s150509610

**Published:** 2015-04-23

**Authors:** Yongbin Qi, Cheong Boon Soh, Erry Gunawan, Kay-Soon Low, Rijil Thomas

**Affiliations:** School of Electrical and Electronic Engineering, Nanyang Technological University, 50 Nanyang Avenue, 639798 Singapore; E-Mails: qiyo0001@e.ntu.edu.sg (Y.Q.); egunawan@ntu.edu.sg (E.G.); ekslow@ntu.edu.sg (K.-S.L.); rijil001@e.ntu.edu.sg (R.T.)

**Keywords:** ultrasound, wireless sensor network, joint angles, squat, rehabilitation, inverse kinematics

## Abstract

This paper presents an unrestrained measurement system based on a wearable wireless ultrasonic sensor network to track the lower extremity joint and trunk kinematics during a squat exercise with only one ultrasonic sensor attached to the trunk. The system consists of an ultrasound transmitter (mobile) and multiple receivers (anchors) whose positions are known. The proposed system measures the horizontal and vertical displacement, together with known joint constraints, to estimate joint flexion/extension angles using an inverse kinematic model based on the damped least-squares technique. The performance of the proposed ultrasonic measurement system was validated against a camera-based tracking system on eight healthy subjects performing a planar squat exercise. Joint angles estimated from the ultrasonic system showed a root mean square error (RMSE) of 2.85° ± 0.57° with the reference system. Statistical analysis indicated great agreements between these two systems with a Pearson's correlation coefficient (PCC) value larger than 0.99 for all joint angles' estimation. These results show that the proposed ultrasonic measurement system is useful for applications, such as rehabilitation and sports.

## 1. Introduction

Squat exercise is an effective training exercise for maintaining mobility and improving lower-limb muscle function [1]. Therefore, it has been included as part of athletic training and rehabilitation. Squat exercise can be investigated using a wide variety of measured and estimated parameters. In [2], the hip and knee torques combined with the position of the knee have been used for the assessment of barbell squat. Ankle, knee and hip joint kinematics have been used in [3] to quantitatively evaluate the motion of squat. In many of these applications, it is essential to monitor the squat exercise under a natural environment without hindrance. Therefore, this entails the need for an unrestrained low-cost motion tracking system.

The most accurate measurement system in capturing the squat motion or other lower trunk movement is the camera-based tracking system, which employs one or more cameras to capture the displacement of reflective markers placed at specific anatomical sites on limb segments [4]. However, such a system requires a complex and expensive experimental setup [5]. Furthermore, it is sensitive to changes in lighting, clutter and shadow. However, a recent development in computer gaming technology, the Microsoft Kinect, is inexpensive, portable and does not require markers to determine anatomical landmarks and, consequently, may overcome the limitations associated with laboratory-based movement analysis systems [6,7]. However, the limitation of the Microsoft Kinect system is the inability to assess internal/external joint rotations in the peripheral limbs [7].

The development of microelectromechanical system technology has produced many low -cost and small inertial sensors, which can be used in a human tracking system, such as an accelerometer and a gyroscope [8–10]. In [11], Brandes et al. use the accelerations measured at the lower trunk to extract spatial-temporal gait parameters with healthy children. Daniel *et al.* [12] proposed an ambulatory measurement system, using a triaxial accelerometer and a dual-axis gyroscope, to assess the vertical displacement of the foot during walking. Although these inertial sensors overcome the shortcomings of a camera-based tracking system, they require double integration to estimate the position and orientation of human motion [13]. Unfortunately, it is difficult to obtain accurate motion accelerations, because of the presence of sensor bias and measurement noise, thus making the displacement error increase exponentially over integration time [12]. This issue can be mitigated either by applying some techniques to correct it periodically, such as zero velocity update (ZUPT), or by applying a Kalman filter [14], or by combining with other sensors, such as imaging sensors, radio frequency identification (RFID) technology or ultra-wide band (UWB) techniques [15–18]. These mentioned hybrid motion tracking systems can improve the tracking accuracy, but with an increased cost, complexity of experiment installation and maintenance.

Since wireless healthcare has received significant interest in recent years, ultra-wideband technology has become a promising technique for general health-monitoring due to its high-temporal resolution and high ranging and positioning accuracy [19]. In [20], wearable UWB transceivers are used to measure some gait parameters (such as the base of support) during the gait. A joint flexion/extension angle measurement system has also been developed in [21,22]. The model is based on providing a high ranging accuracy (intersensor distance) between a pair of UWB transceivers placed on the adjacent segments of the joint center of rotation. The measured distance is then used to compute the joint angles based on the law of cosines. However, in an impulse radio (IR) UWB system, where a sub-nanosecond pulse is used, the required synchronization is extremely challenging. A highly accurate synchronization clock that provides precise timing information is required for tracking, which is difficult to achieve in low-cost systems [23]. Furthermore, it is difficult to sample the received signal in real time with current ADC technology, due to the large bandwidth of the IR-UWB pulse [24].

An alternative method to UWB technology is to use the ultrasound technique due to its low cost, safety, simplicity and high temporal resolution for low range measurement [25]. There are two types of ultrasonic transceivers: one is with an ultrasonic transmitter and receiver on the same circuit board as the one used in [26]. The distance measurement of such an ultrasonic sensor is the returned distance reflected from the ground/surface, and the orientation of the sensor is not taken into consideration. The other type is with an ultrasonic transmitter and receiver on separate boards [27]. However, there are only two receivers used in [27], which only measures one directional displacement.

In this paper, a wireless wearable sensor system based on ultrasound for monitoring squat exercise is proposed. Only one ultrasonic sensor (transmitter) is needed to be attached to the human trunk, which minimizes the discomfort for users and avoids complex calibration procedures and synchronization issues. Several receivers (anchors) with known positions are used to define a coordinate reference system. The system measures propagation delay from anchor to transmitter, thus computing the range in terms of the speed of sound. Then, the range information defines a circle centered at this anchor with a radius equal to the measured distance, and the position of the transmitter should be within the intersection of several such circles. This is called the spherical tracking technique [28]. The proposed approach makes use of the wireless sensor network concept with all of the mobile sensor nodes wirelessly communicating with the coordinator, thus eliminating the usage of wires. In addition, the sensor node attached to the human trunk is light and small. Furthermore, it is low cost compared to the camera system.

The paper is organized as follows: Section 2 gives a brief overview of the configuration of the wireless wearable system. The biomechanical model of the leg, the displacement measurement model and the inverse kinematic model using the damped least-squares technique are given in Section 3. Section 4 investigates the performance of our ultrasonic tracking system by comparing with the camera-based tracking reference system. Finally, the discussion and conclusion are given in Section 5.

## 2. Materials

**Figure 1:**
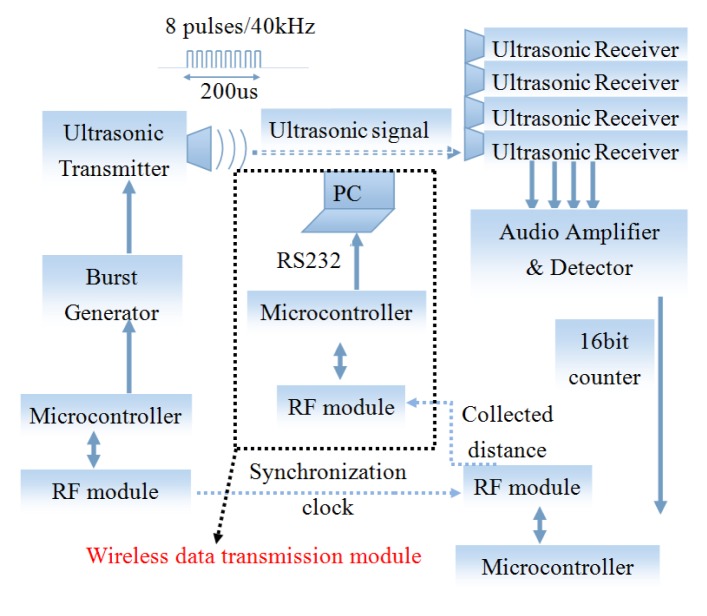
General configuration of the system.

Figure 1 shows the general configuration of the proposed system. The ultrasonic measurement system, used in our previous work [29,30], is comprised of one ultrasonic transmitter (mobile) and four ultrasonic receivers (anchors). The mobile node is composed of a circuit board (form factor: 4 cm × 6 cm) with a microcontroller and a radio frequency (RF) module, along with a separate circuit board (form factor: 4 cm × 3 cm × 1.6 cm) with an ultrasound generator. The displacements of the ultrasonic transmitter measured using the spherical tracking technique were expressed in a global coordinate system, which described trunk position relative to the ground. The *X*-axis was defined as the direction of anterior-posterior direction; the *Y*-axis was made to be in the vertical direction. The third axis of the coordinate system, the *Z*-axis, was determined in such a way, so as to form a right-handed coordinate system.

In the proposed system, ultrasonic range measurements are initiated by a periodic trigger input with a pulse duration of 10 μs. Then, the ultrasound transmitter is triggered to produce an ultrasonic burst consisting of 8 pulses with a frequency of 40 kHz. Meanwhile, the RF module on the mobile node is triggered synchronously, thus sending out a data package with the timer starter command (TSC) using the broadcast address to notify the anchors that an ultrasound signal has been transmitted. Once the anchor receives the TSC, it will start its 16-bit counters to record the propagation delay from the mobile to the anchor. The transmission time of the RF signal from the mobile is negligible, since the speed of light is much faster than the speed of ultrasound. The 16-bit counter will stop counting immediately after each of the transmitted bursts is detected by the anchor. Then, the counted steps will be converted to propagation delay by multiplying the time resolution (instruction cycle) of the microcontroller. The device uses the STC12LE5612AD microprocessor with high crystal frequency (Fosc = 22.1184 MHz), which provides high time resolution, resulting in a theoretical resolution of 0.031 mm (340 m/s /(Fosc/2)). From this delay, the distance between the mobile and the anchor can be calculated using:

(1) $$d=t\cdot \upsilon_{s}$$

where *d* is the distance in meters, *t* is the propagation delay in seconds and *υ_s_* is the speed of ultrasound in air. The ultrasound velocity can be approximated to:

(2) $$\upsilon_{s}=331.5+0.6T_{c}$$

where *T_c_* is air temperature in degrees Celsius. After collecting all four distances from the transmitter to anchors, the coordinator node forwards all of the collected information to a wireless data transmission module, and then, it is transmitted to a personal computer through an RS232 cable for post-processing.

## 3. Methods

In this section, we discuss our proposed angle tracking algorithm. We first construct the leg kinematic model using a set of links connected by joints. Then, the Denavit-Hartenberg parameters of the leg model are computed. Horizontal and vertical displacement of the ultrasonic sensor are obtained from the data recorded with a single ultrasound transmitter placed at the center line of the torso using an extended Kalman filter. These displacements, together with known joint constraints during squatting, are then used as input to a damped least-squares-based inverse kinematic model, thus allowing the determination of the corresponding ankle, knee and hip joint flexion/extension angles.

### 3.1. Biomechanics of the Human Body

The commonly-used method for modeling a human rigid body is based on a sequence of links connected by joints. This model could better represent the movement of any parts on the human body [31]. In order to describe the position and orientation between adjacent links, Denavit and Hartenberg (D-H) in 1955 proposed a systematic notation for assigning right-handed orthogonal coordinate frames to each link [32]. The transformations between two adjacent frames can be described by a 4 × 4 homogeneous transformation matrix. Then, the transformation is described by four parameters associated with each link, known as D-H parameters (***L****_k_* = (*θ_k_, d_k_*, *a_k_*, *α_k_*)). The first one is the joint angle *θ_k_*, which is defined as the angle between *x^k^*^−1^ and *x^k^* axes about the *z^k^*^−1^ axis. The second parameter is *d_k_*, which is the distance between *x^k^*^−1^ and *x^k^* axes along the *z^k^*^−1^ axis. The third one is *a_k_*, which is defined as the distance between *z^k^*^−1^ and *z^k^* axes along the *x^k^* axis. The last parameter is *α_k_*, which is the twist angle between *z^k^*^−1^ and *z^k^* axes about the *x^k^* axis.

**Figure 2:**
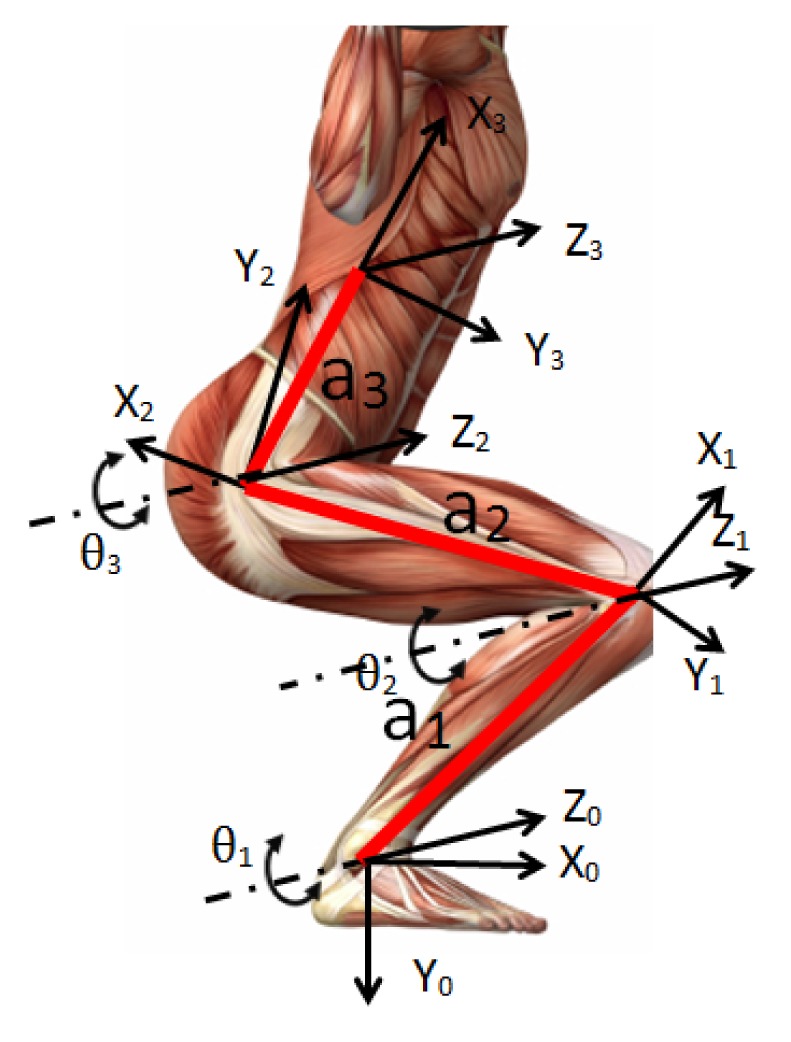
Three degrees-of-freedom kinematic model of the leg.

Figure 2 and Table 1 show the biomechanical model of the human body used in our study and D-H parameters for the leg kinematics, respectively, which correspond to a kinematic chain comprised of three rigid segments (shank (*a*_1_), thigh (*a*_2_) and torso (*a*_3_)).

### 3.2. Determination of Orientation and Position

Given the D-H model and parameters presented before, we then proceed to investigate the orientation and position of the sensor point, where the ultrasonic sensor will be attached. The orientation *R* and position ***p*** = [*x y z*]*^T^* of the torso (or ultrasonic sensor) can be calculated with respect to the ankle frame as follows:

(3) $$\begin{array}{l} \text{R}=\left[\begin{array}{ccc} C\theta_{123} & -S\theta_{123} & 0\\ S\theta_{123} & C\theta_{123} & 0\\ 0 & 0 & 1 \end{array}\right]\\ \text{p}=\left[\begin{array}{c} a_{3}C\theta_{123}+a_{2}C\theta_{12}+a_{1}C\theta_{1}\\ a_{3}S\theta_{123}+a_{2}S\theta_{12}+a_{1}S\theta_{1}\\ 0 \end{array}\right] \end{array}$$

where the orientation angle *ϕ* = *θ*_123_ = *θ*_1_ + *θ*_2_ + *θ*_3_ and *θ*_12_ = *θ*_1_ + *θ*_2_. *θ*_1_, *θ*_2_ and *θ*_3_ are the ankle, knee, and hip joint angles, respectively.

### 3.3. Determination of the Ultrasonic Sensor Position

In most cases, it is expected to minimize the number of sensors attached to the human body, not only for unrestrained movement or financial cost, but also for simplifying the calibration process and synchronization issues [1,33]. Therefore, only one mobile target is used in our measurement system. As mentioned in Section 2, we assume that the mobile lying on the position ***p*** sends out ultrasound signals to the anchors during the discrete sampling time. The positions of the anchors are known to be ***p****_i_* = [*x_i_ y_i_ z_i_*]*^T^*, respectively After receiving all of the measurements, the coordinator will compute an estimate of the state (position and velocity) of the mobile using extended Kalman filter (EKF) estimation. We will explain the tracking algorithm in more detail later.

#### 3.3.1. Mobile Target Motion Model

We design a state vector with six components, three Cartesian coordinates ***p*** = [*x y z*]*^T^* and three velocity components along the three coordinate axes ***ṗ*** = [*ẋ ẏ ż*]*^T^*, to describe the mobile moving in a 3-dimensional space. In other words, the state of the mobile target on time step *k* can be expressed as:

(4) $$\text{x}\left(k\right)={\left[x\left(k\right)\;y\left(k\right)\;z\left(k\right)\;\dot{x}(k)\;\dot{y}(k)\;\dot{z}(k)\right]}^{T}$$

Then, the following model is used to describe the motion of the mobile:

(5) x(k)=Ax(k−1)+Bω(k−1)

where:

(6) $$\begin{array}{cc} A=\left[\begin{array}{cc} I_{3\times 3} & T\cdot I_{3\times 3}\\ O_{3\times 3} & I_{3\times 3} \end{array}\right], & B= \end{array}\left[\begin{array}{c} \frac{T^{2}}{2}\cdot I_{3\times 3}\\ T\cdot I_{3\times 3} \end{array}\right]$$

where *I*_3×3_ indicates the identity matrix and *O*_3×3_ indicates the zero matrix. *T* is the sampling interval between time step *k* and *k* − 1. *ω*(*k* − 1) = [*ω_x_ ω _y_ ω_z_*]*^T^* is a white Gaussian noise with zero mean and covariance matrix $Q_{\omega }=\text{diag}\left(q_{x}^{2},q_{y}^{2},q_{z}^{2}\right)$. In most cases, *q_x_, q_y_* and *q_z_* can be considered as standard deviations of the velocity noise along the *x*-, *y*- and *z*-axes, respectively.

#### 3.3.2. Measurement Model

At time step *k*, let *d_i_*(*k*) denote the absolute distance measured at the *i*-th anchor using the following equation:

(7) $$d_{i}=\left\|\text{p}-{\text{p}}_{i}\right\|+{\tilde{d}}_{i}$$

where *d̃_i_* is the range measurement error and *d_i_*(*k*) is simplified to *d_i_*. Stacking all of the distance information, we have the measurement model expressed as:

(8) $$D\left(k\right)=F\left(\text{x}\left(k\right)\right)\text{x}\left(k\right)+\tilde{D}\left(k\right)$$

where:

(9) $$\begin{array}{c} D\left(k\right)={\left[\begin{array}{cccc} d_{1} & d_{2} & \cdots & d_{n} \end{array}\right]}^{T}\\ F\left(\text{x}\left(k\right)\right)={\left[\begin{array}{cccc} F_{1} & F_{2} & \cdots & F_{n} \end{array}\right]}^{T}\\ F_{i}\left[\begin{array}{cccccc} \frac{\partial d_{i}}{\partial x} & \frac{\partial d_{i}}{\partial y} & \frac{\partial d_{i}}{\partial z} & 0 & 0 & 0 \end{array}\right]\\ \tilde{D}\left(k\right)={\left[\begin{array}{cccc} {\tilde{d}}_{1} & {\tilde{d}}_{2} & \cdots & {\tilde{d}}_{n} \end{array}\right]}^{T} \end{array}$$

*n* is the number of anchors, and *D̃*(*k*) ∼ *N*(*0,R*(*k*)) are the measurement errors. $R\left(k\right)=\text{diag}\left(e_{1}^{2},e_{2}^{2},\cdots ,e_{n}^{2}\right)$ is the covariance matrix of measurement errors. *e_i_* is always considered as the standard deviation of the measurement error of anchor *i*.

#### 3.3.3. Tracking Using the Extended Kalman Filter

The basic idea of the extended Kalman filter is that the filter uses prior knowledge of all distance information to predict and produce an estimate of the position for where the mobile might be in the next time step. Once the next distance sample arrives, the filter first corrects the state based on the actual distance information [34,35]. In each iteration, the current state ***x***(*k* − 1) is used to predict the velocity and position at the time step *k* − 1. The error covariance *P̂*(*k*) is also predicted using the state space model in Equation (5). This is followed by the computation of the Kalman filter gain *K*(*k*). Once *K*(*k*) is computed, the range measurements *D*(*k*) are calibrated by the predicted state of the mobile and are used to update the mean and covariance of the state ***x***(*k*) and *P*(*k*), respectively. The standard expression is given below [36]:

- Prediction:
  (10) $$\begin{array}{l} \hat{\text{x}}\left(k\right)=Ax\left(k-1\right)\\ \hat{P}\left(k\right)=AP\left(k-1\right)A^{T}+BQ_{\omega }B^{T} \end{array}$$
- Update:
  (11) $$\begin{array}{ll} \Theta \left(k\right) & =D\left(k\right)-F\left(\hat{\text{x}}\left(k\right)\right)\hat{\text{x}}\left(k\right)\\ S\left(k\right) & =F\left(\hat{\text{x}}\left(k\right)\right)\hat{P}\left(k\right)F^{T}\left(\hat{\text{x}}\left(k\right)\right)+R\left(k\right)\\ K\left(k\right) & =\hat{P}\left(k\right)F^{T}\left(\hat{\text{x}}\left(k\right)\right)S{\left(k\right)}^{-1}\\ \text{x}\left(k\right) & =\hat{\text{x}}\left(k\right)+K\left(k\right)\Theta \left(k\right)\\ P\left(k\right) & =\hat{P}\left(k\right)-K\left(k\right)S\left(k\right)K{\left(k\right)}^{T} \end{array}$$

Once the state of the mobile ***x***(*k*) is computed at time step *k*, the position information is used to obtain the joint angles when subjects are doing the squat exercise. Since the displacement along lateral direction does not change, the squat exercise is always planar. Therefore, in the rest of our paper, only horizontal and vertical displacement (Cartesian coordinates) are considered as input to the inverse kinematic model.

#### 3.4. Inverse Kinematic Model

The direct kinematic mapping of interest for the system is given by:

(12) $$\begin{array}{ll} \text{p} & =f\left(\theta \right)\\ \dot{\text{p}} & =J\left(\theta \right)\dot{\theta } \end{array}$$

where ***θ*** = [*θ*_1_ *θ*_2_ *θ*_3_]*^T^*, ***θ̇*** and ***ṗ*** are joint velocities and Cartesian velocities, respectively, *f* is the nonlinear function described in Equation (3) and *J* is a Jacobian matrix defined as $J\left(\theta \right)={\left[\frac{\partial f}{\partial \theta_{1}}\frac{\partial f}{\partial \theta_{2}}\frac{\partial f}{\partial \theta_{3}}\right]}^{T}$. The system under study is kinematically redundant during a planar squat exercise, since there is a 3-degrees-of-freedom (DoF) movement with only 2 displacements known.

A general solution in terms of a generalized inverse of the Jacobian matrix is [37,38]:

(13) $$\dot{\theta }=ϝ\left(p-{\hat{p}}_{e}\right)+\left(I-ϝJ\right)\Phi$$

where ***p̂****_e_* is the estimated position obtained from the forward kinematic model and ***p*** is the Cartesian coordinates (target position) acquired by the ultrasonic sensor using EKF. ***ϝ*** is a generalized inverse matrix of the Jacobian matrix, (*I* − ***ϝ J***) is the matrix that projects the vector Φ in the null space of *J*, *J*(*θ*) is simplified to *J* and:

(14) $$\Phi =\xi_{0}{\left(\frac{\partial W\left(\theta \right)}{\partial \theta }\right)}^{T}$$

where *ξ*_0_ > 0 and *W*(***θ***) is a scalar (objective) function of joint variables. Since the solution moves along the direction of the gradient of *W*(***θ***), it attempts to locally maximize subject to the kinematic constraint. The objective function is based on a limited joint range from lower ***θ****_min_* to upper ***θ****_max_* limits, shown in Table 2 [1]:

(15) $$\begin{array}{l} W\left(\theta \right)=-\frac{1}{6}{\overset{3}{\underset{i=1}{\text{∑}}}\left(\frac{\theta_{i}-{\bar{\theta }}_{i}}{\theta_{i\max}-\theta_{i\min}}\right)}^{2}\\ {\bar{\theta }}_{i}=\frac{\left(\theta_{i}\max+\theta_{i}\min\right)}{2} \end{array}$$

where *θ̄_i_* is the middle value of the joint range; thus, redundancy is exploited to keep the joint variables ***θ*** as close as possible to the center of their ranges [39].

#### 3.5. Damped Least-Squares Scheme

The system is close to a singular point near/at the beginning and ending of the squat cycle, since the Jacobian matrix does not have full rank [40]. The unavoidable measurement errors in displacement estimations using the EKF algorithm will produce some unreachable targets, which leads to oscillations and shaking of the inverse kinematic problem. An alternative solution overcoming the problem of inverting differential kinematics in the neighborhood of a singularity is damped least-squares inverse (DLS):

(16) $$ϝ=J^{T}{\left(JJ^{T}+\kappa^{2}I\right)}^{-1}$$

where *κ* is a damping factor that ensures continuity and good shaping of the solution near/at singularity and for out-of-reach targets. The damping factor should be critically determined for obtaining good performance over the entire workspace as follows:

(17) $$\kappa^{2}=\{\begin{array}{ll} 0 & \begin{array}{cc} \text{when} & \sigma_{\text{min}}\geq \varepsilon \end{array}\\ \left(1-{\left(\frac{\sigma_{\text{min}}}{\varepsilon }\right)}^{2}\right)\kappa_{\max}^{2} & \text{otherwise} \end{array}$$

where *κ_max_* is used to adjust the solution in the neighborhood of a singularity, *σ_min_* is the estimate of the smallest singular value and *ε* is defined as the size of the singular region [41].

To gain more insight into the features of the DLS, the singular value decomposition [40,41] of the Jacobian matrix is introduced, which is:

(18) $$J=\sum_{i=1}^{3}\sigma_{i}{\text{u}}_{i}\upsilon_{i}^{\text{T}}$$

where ***u****_i_* and ***υ****_i_* are the output and input singular vectors and *σ_i_* are the singular values. Thus, the solution of DLS can be expressed in the form of:

(19) $$F=J^{\text{T}}{\left(JJ^{T}+\kappa^{2}I\right)}^{-1}=\sum_{i=1}^{3}\frac{\sigma_{i}}{\sigma_{i}^{2}+\kappa^{2}}\upsilon_{i}{\text{u}}_{i}^{T}$$

From Equation (19), the components for which *σ_i_* ≫ *κ* are little affected by the damping factor, since for large *σ_i_*, $\sigma_{i}/\left(\sigma_{i}^{2}+\kappa^{2}\right)\approx 1/\sigma_{i}$. On the contrary, when *σ_i_* is of a similar or even smaller magnitude as *κ*, then the associated component of the solution is driven to zero by the factor *σ_i_*/*κ*^2^. Therefore, the DLS method tends to effectively smooth out the performance in the neighborhood of singularities.

Once the solution of Equation (13) is found, the joint angles can be initially computed using previous joint angles and the estimated joint velocities:

(20) $$\theta_{k+1}=\theta_{k}+\dot{\theta }$$

The above joint angles estimation can be further refined iteratively: For *j* = 0, 1, ⋯, compute:

(21) $$\begin{array}{ll} {\text{p}}_{k+1}^{j+1} & =f\left(\theta_{k+1}^{j}\right)\\ {\dot{\theta }}_{k+1}^{j+1} & ={ϝ}_{k+1}^{j}\left(\text{p}-{\hat{\text{p}}}_{k+1}^{j+1}\right)+\left(I-{ϝ}_{k+1}^{j}J\right)\Phi_{k+1}^{j} \end{array}$$

until:

(22) $$\left\|\text{p}-{\text{p}}_{k+1}^{j+1}\right\|\leq \xi$$

and *ξ* ≥ 0 is a predefined threshold.

#### 3.6. Parameters Identification

##### 3.6.1. Noise Statistics in the Kalman Filter

Using the measurement model in Equation (7), we first conduct a static measurement to estimate the covariance matrix of measurement error, *R*(*k*). It is reasonable to assume that all of the sensors have independent distributed noise. We take a sensor and run *M* tests with different distances between the receiver and transmitter. The actual distance for test *i, r_i_*, is known, and there are *N* measurement samples $m_{i}^{j}$ collected for each *r_i_*, where *j* = 1, ⋯, *N*.

Therefore, straightforward calculations lead to the estimation of the mean and variance of the measurement errors:

(23) $$\begin{array}{ll} u & =\frac{1}{MN}\overset{M}{\underset{i=1}{\text{∑}}}\overset{N}{\underset{j=1}{\text{∑}}}\left(m_{i}^{j}-r_{i}\right)\\ e^{2} & =\frac{1}{M\left(N-1\right)}\overset{M}{\underset{i=1}{\text{∑}}}\overset{N}{\underset{j=1}{\text{∑}}}{\left(m_{i}^{j}-u\right)}^{2} \end{array}$$

##### 3.6.2. Parameter Identification of the Inverse Kinematic Model

The damping factor is computed in (17) with *ε* = 0.04 and *κ_max_* = 0.04. These values were determined by calculating the smallest singular value among all of the near singular Jacobian matrices *J*, where the subjects under test were close to an upright posture.

### 4. Experimental Validation

#### 4.1. Experimental Setup

**Figure 3:**
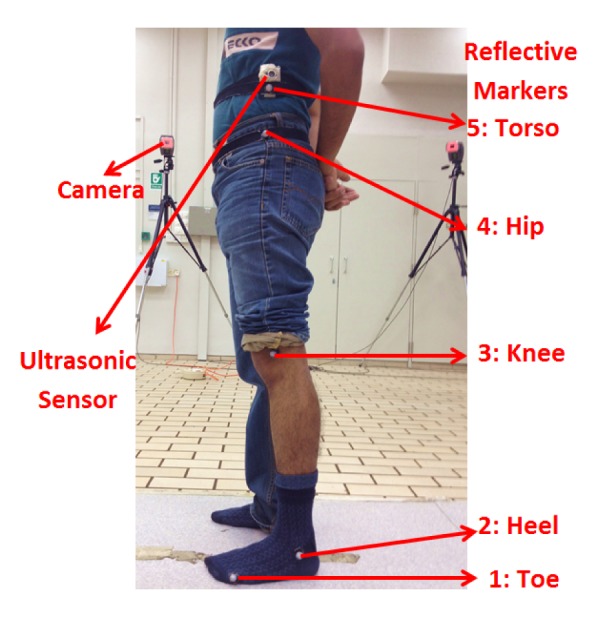
Wireless unit with embedded ultrasonic sensor placed on the lateral side of the trunk and five reflective markers fixed on the toe, heel, knee, hip and torso for the optical tracking reference system.

To compare the performance of the proposed measurement system with a conventional measurement system, experiments were conducted in a motion analysis lab with eight high speed cameras in the School of Mechanical and Aerospace Engineering, Nanyang Technological University. Eight healthy subjects (mean age 24.5 ± 1.73 years, mean weight 61.3 ± 6.40 kg, mean height 171.8 ± 5.80 cm) volunteered in the experiments. All subjects were required to wear a ultrasound transmitter on the torso and five reflective markers, as shown in Figure 3, to perform an unrestrained squat exercise at a self-selected speed, keeping their feet flat on the ground. Each subject was asked to repeat the squat exercise five times. The segment length and joint angles with respect to the kinematic model in Figure 2 were calculated using the instantaneous positions of the above-mentioned five reference markers.

There were four anchors used in our experiment with positions (***p***_1_ = [0 0 0]*^T^*, ***p***_2_ = [0.324*m* 0 0]*^T^*, ***p***_3_ = [0.324*m* 0.230*m* 0]*^T^*, ***p***_4_ = [0 0.230m 0]*^T^* ). In our method, only one ultrasonic sensor (transmitter) is needed, which is to be attached to the foot, which minimizes the discomfort for users and avoids complex calibration procedures and synchronization issues. All of the data transmission between anchors, coordinator and transmitter are done wirelessly through the RF module. Therefore, it does not restrict the movement of the subjects.

The synchronization between the proposed ultrasonic and reference camera measurement system was done by maximizing the correlation between the horizontal displacements of the torso point estimated by both systems. The proposed ultrasonic sensor data were acquired at 50 Hz. Data from the reference system were acquired at 200 Hz. All data were low-pass filtered by a second order low-pass Butterworth filter at 5 Hz. The positions of ankle, knee, hip and torso markers and related joint angles estimated from the motion capture system were considered as reference data and used for the evaluation of the ultrasound-based system.

#### 4.2. Data Analysis

To verify the joint angle estimation accuracy, which is implemented in the hardware platform, as described in Section 2, the mean and standard deviation value of the difference between the parameters extracted with the optical tracking reference system, together with root mean square error (RMSE), were computed. Pearson's correlation coefficient (PCC) is also used in gauging the degree of agreement between these two systems. Furthermore, a statistical method, Bland-Altman analysis [42], for assessing the level of agreement between the proposed and reference systems was applied. The limits of agreement refer to the mean ± 1.96-times the standard deviation of the difference between the two systems.

#### 4.3. Results

**Figure 4:**
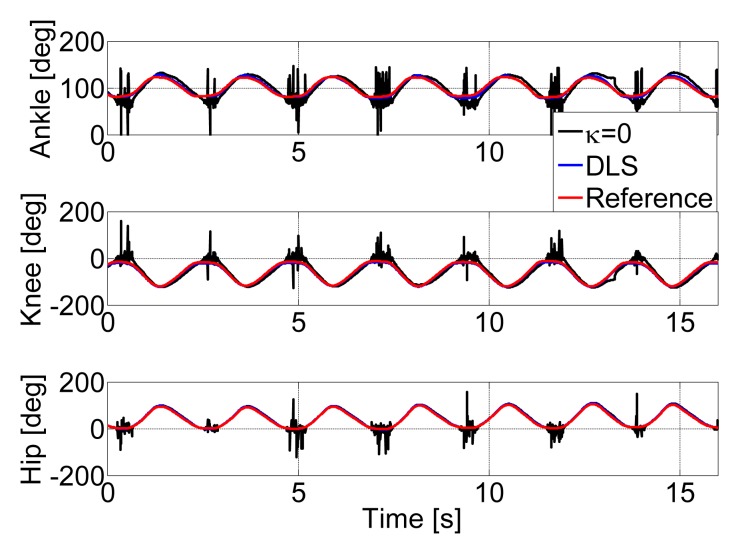
The joint angles obtained from both ultrasonic and optical tracking system during a 15-s consecutive planar squat exercise. The black line makes use of the pseudo-inverse method, that is *κ* = 0. The blue line is the damping least-squares method. The red line is a reference result.

In Figure 4, a randomly selected trial for the estimated ankle, knee and hip joint flexion and extension angles during a squat exercise is shown. Figure 4 shows the effect of different damping factors on the joint angle estimation. We compared with the pseudo-inverse method (*κ* = 0) and DLS method. It is shown clearly that the pseudo-inverse method performs poorly because of instability near singularities (*σ_i_* approaches zero). However, the DLS method tends to act similarly to the pseudo-inverse method away from singularities and effectively smooths out the performance of the pseudo-inverse method in the neighborhood of singularities. All of the joint angles estimated by the DLS method show a great correspondence with the reference system.

**Figure 5:**
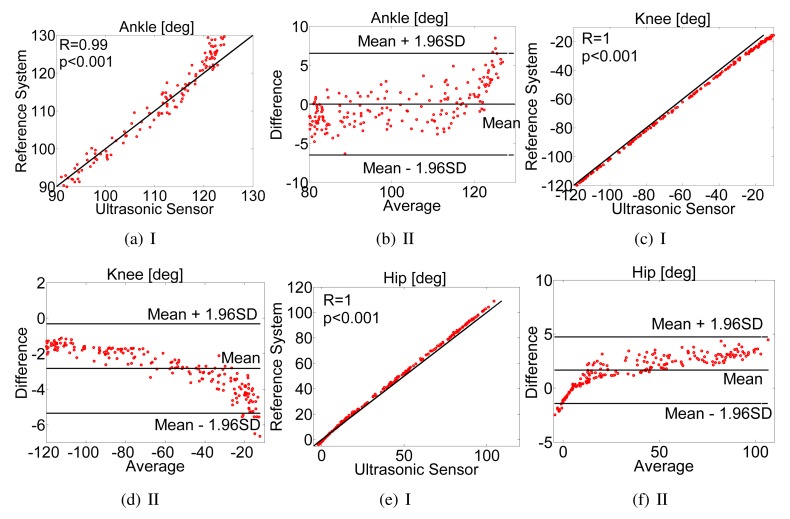
(I) Joint angles estimated by the proposed ultrasonic and reference camera-based measurement system, plotted with the line of equality. (**a**) Ankle joint angle; (**c**) knee joint angle and (**e**) hip joint angle. (II) Bland–Altman plot with the mean and difference between two values estimated by the proposed ultrasonic and reference camera-based measurement system, plotted with the limits of agreement, which are the average difference ± 1.96-times the standard deviation of the difference. (**b**) Ankle joint angle; (**d**) knee joint angle and (**f**) hip joint angle.

For the clarity of the Bland–Altman plots, we randomly selected 4 trials to draw these figures, as shown in Figure 5. The data plotted in Figure 5I show that all points lie along the line of equality, which means a high degree of agreement between the measurements. The PCC values between the two system are also calculated for the data in Figure 5I. For all of these joint angles, the correlations are all equal to or larger than 0.99 (*p* < 0.001), where the *p*-value is the probability that the measurements by the two methods are not linearly related. As the *p*-value is extremely small, we can safely conclude that the measurements of joint angles by these two methods are significant. Figure 5II shows the Bland-Altman plot for the selected data by plotting the difference between the two systems against the mean values of ankle, knee and hip joint angles. This plot is used to gauge the limits of agreement between the two methods. The 95% confidence intervals were determined to be (−6.48°, 6.56°) for ankle angles, (−5.34°, −0.32°) for knee angles and (−1.42°, 4.7°) for hip angles.

All of the collected data from 8 subjects are used to compute the mean and standard deviation of the difference, RMSE, PCC values and the limits of agreement, which are reported in Table 3. Horizontal displacement was obtained with a difference of 1.08 ± 6.07 mm, an RMSE of 6.50 ± 2.08 mm and a high PCC value of 0.94 ± 0.04. The mean difference and standard deviation of the vertical displacement was 0.75 ± 9.65 mm, which had a high similarity of 0.94 ± 0.06 with the results of the reference system. For all of the collected data, the joint angles of interest were estimated with a mean difference from the reference data of 2.85° (RMSE) and with high similarities greater than 0.99, where all *p*-values are smaller than (0.001). For all three estimated joint angles, the hip joint has the smallest error, and consequently, tighter lower and upper limits of agreement, closer to zero than the ankle and hip joint. In summary, the great agreement of all joint angles estimated by the two systems is enough for us to be confident that the proposed measurement system can be used for further clinical applications.

### 5. Discussion and Conclusions

This study has demonstrated that one ultrasound transmitter (attached to the human body) and four receivers can be used to obtain high accuracy ankle, knee, hip joint flexion/extension angles estimates. The extended Kalman filter is applied to estimate the displacements in the vertical and horizontal direction of the ultrasonic sensor, and then, the recorded displacements together with known joint constraints are used to estimate the joint angles of the trunk using the damped least-squares-based technique for the singularity avoidance problem of redundant systems.

The proposed measurement system was compared to a camera-based system, and the results were averaged over eight subjects. These metrics (difference, RMSE, PCC values and limits of agreement) are shown in Table 3 to illustrate the differences between these two systems. The experimental results showed that the horizontal and vertical displacement of the ultrasonic sensor and the joint angles (hip, knee and ankle) can be estimated with relatively small differences. Bland–Altman analysis indicated a great agreement between these two systems, with the PCC value higher than 0.99. The average RMSD of the estimate of the lower extremity joint angles is 2.85° ± 0.57°, which is similar to what has been obtained in other studies using inertial sensors to estimate these joint angles [1,13].

Although the positive results showed the feasibility of applying such a system for in-home monitoring, there is an issue to be addressed in further research, that is how to deal with the multipath propagation. All of the experiments in this study are conducted under the line-of-sight condition, where the ultrasonic transmitter faces all of the receivers without any obstacles between them. Therefore, for 3D displacements, according to the spherical positioning technique, a minimum of four anchors with known positions is required. The method used in our experiment to mitigate the multipath propagation is by setting an inhibition time, *i.e.*, the ultrasound detector will be disabled within the inhibition time to detect an ultrasound signal. Then, it will be enabled after this inhibit duration. Another possible solution is that we can use more receivers, which can not only account for multipath propagation, but also increase the measurement volume and accuracy of the proposed system [19].

A key issue in the system is the determination of the damping factor in Equation (16), since it plays an important role in finding a solution to the inverse kinematic model. Larger *κ* makes the solution for joint velocities behave well in the neighborhood of a singularity, but results in low tracking accuracy and a low convergence rate. Small values of the damping factor give accurate solutions, but not are robust to the singular or near-singular region. Hence, it is essential to select suitable values for the damping factor.

In summary, this paper presents an unrestrained measurement system based on a wearable wireless ultrasonic sensor network to track the lower limb joint and trunk kinematics during a squat exercise with only one ultrasonic sensor attached to the trunk. The camera-based tracking reference system was used to benchmark the performance of the proposed ultrasonic motion analysis system. It demonstrates acceptable accuracy for further applications. Additionally, the proposed system is easy to wear, to use and much less expensive than current camera systems. It does not restrict the movement of patients or subjects with bulky cables.

## Figures and Tables

**Table 1:** Denavit–Hartenberg parameters of the leg model.

**Joint**	***θ****_k_*	***d****_k_*	***a****_k_*	***α****_k_*
Ankle (*L*_1_)	*θ*_1_	0	*a*_1_	0
Knee (*L*_2_)	*θ***_2_**	0	*a*_2_	0
Hip (*L*_3_)	*θ***_3_**	0	*a*_3_	0

**Table 2:** Range of motion and constraints imposed in the system.

	**Upper Limit (deg)**	**Lower Limit (deg)**
*θ*_1_ ankle joint	150	80
*θ*_2_ knee joint	5	−120
*θ*_3_ hip joint	110	−15

**Table 3:** Comparison of errors in measurements for the proposed and reference system. The mean difference and relevant standard deviation (std) together with Pearson's correlation coefficient (PCC) values are shown. Bland–Altman limits of agreement are also reported. HD: Horizontal Displacement; VD: Vertical Displacement.

	**Difference**		**RMSE**		**PCC**		**Limits of Agreement**	
			
	**Mean**	**Std**	**Mean**	**Std**	**Mean**	**Std**	**Lower**	**Upper**
HD (mm)	1.08	6.07	6.50	2.08	0.94	0.04	−10.82	12.97
VD (mm)	0.75	9.65	9.90	4.79	0.94	0.06	−18.17	19.67
ankle (deg)	0.57	3.10	3.10	1.10	0.99	0.01	−6.07	6.07
knee (deg)	−2.46	1.82	3.28	0.39	1.00	0.00	−6.03	1.11
hip (deg)	1.49	1.56	2.18	0.21	1.00	0.00	−1.55	4.54
